# Safety and efficacy of pilocarpine, cevimeline, and diquafosol compared to artificial tears for the treatment of dry eye: protocol for a systematic review

**DOI:** 10.1186/s13643-022-01979-4

**Published:** 2022-05-28

**Authors:** José Gerardo Serrano-Robles, Ana Karen Pérez Vázquez, Alejandro Navas, Enrique O. Graue-Hernandez, Arturo Ramirez-Miranda, Nicolás Kahuam-López

**Affiliations:** 1 Centro de Investigación en Ciencias de La Salud (CICSA), Facultad de Ciencias de La Salud, Universidad Anáhuac México, Campus Norte, Mexico City, Huixquilucan Mexico; 2 Cornea and Refractive Surgery Department, Instituto de Oſtalmología Fundación Conde de Valenciana IAP, Mexico City, Mexico

**Keywords:** Systematic review, Meta-analysis, Protocol, Secretagogues, Artificial Tears, Dry eye

## Abstract

**Background:**

Dry eye disease (DED) is a condition that compromises the ocular surface and affects millions of people around the world. In recent years, a scheme has been proposed for the treatment of DED, with the use of artificial tear being the mainstay of treatment. In this scheme, the use of secretagogues is suggested as part of the treatment for patients with moderate to severe affectation. With this systematic review, we aim to evaluate the effectiveness and safety of secretagogues for DED.

**Methods:**

Electronic databases will be searched; we will include randomized controlled trials that compare secretagogues and artificial tears. Study inclusion will not be restricted on the basis of language or publication status. We will use Google Translate to assess studies written in languages other than English and Spanish. Identification, evaluation, data extraction, and assessment of risk of bias will be conducted by two authors of the review, a third review author will resolve any disagreement. The outcomes will be the ocular surface disease index score, tear film break-up time, Schirmer test score, VRQoL Score, and tear film osmolarity. We will use the Cochrane Collaboration Risk of Bias 2 (RoB 2) tool for assessing the risk of bias of the included studies.

Based on the heterogeneity of the included studies, we will combine the findings in a meta-analysis using a fixed effect model if heterogeneity ≤ 50% or a random effect model if heterogeneity > 50%. If we deem meta-analysis as inappropriate, we will document the reasons and report findings from the individual studies narratively.

**Discussion:**

Based on the evidence obtained, we will evaluate the effect of pilocarpine, cevimeline, and diquafosol and compare it to artificial tears on multiple outcome measures.

This systematic review aims to determine the efficacy and safety of the secretagogues pilocarpine, cevimeline, and diquafosol to help clinicians in the decision-making process.

**Trial registration:**

PROSPERO CRD42020218407.

**Supplementary Information:**

The online version contains supplementary material available at 10.1186/s13643-022-01979-4.

## Background

### Description of the condition

Dry eye disease (DED) is one of the main reasons for consultation with ophthalmologists in the clinical setting; it frequently presents with foreign body sensation, burning, and pain associated with blurred vision that negatively impacts the quality of life of patients. It is estimated that the prevalence ranges from 5 to 35% with a predominance of females and with a maximum peak at age 60 where the prevalence reaches 70% with a greater trend for the Asian population [1–5].

The three main components of tear film are the mucin layer, the aqueous layer, and the lipid layer. Different conditions that affect one or more components of the tear film or the glands that produce its components has the potential to produce the disease [4]. Tear hyperosmolarity is considered to be the trigger for a cascade of signaling events within corneal epithelial cells, leading to the release of inflammatory mediators and proteases [6].

The Ocular Surface Disease Index (OSDI) consists of 12 questions and is an instrument designed to provide an effective way to assess ocular surface disease related to dry eye and to estimate the severity of the disease along with its effect on the functional capacity of the patient. In this way, the OSDI allows a reliable diagnosis of dry eye and can be used as a tool to measure the effectiveness of a specific treatment for dry eye disease [7, 8]. The time from the ascending trace of the last blink to the breaking of the tear film or the formation of a dry spot is recorded as the tear break-up time (TBUT) measurement. [7, 9, 10]. The osmolarity of the tear film indicates the balance of inputs and outputs of the lacrimal system. The cut-off points for making the diagnosis of DED by osmolarity is ≥ 308 mOsm/L in one or both eyes or a gradient ≥ 8 mOsm/L in the osmolarity between both eyes [11, 12].

### Description of the intervention

The management of DED is complicated, due to its multifactorial etiology. The goal of treating DED is to restore homeostasis to the ocular surface and tear film, breaking the vicious cycle of the disease.

Management algorithms are often constructed to recommend a sequence of treatments according to the stage of the disease, but this construction is complicated in DED, as the disease often varies from patient to patient, both in severity and in character. In recent years, a scheme has been proposed for the treatment of DED [13], with the use of artificial tears being the mainstay of treatment. In this scheme, the use of secretagogues is suggested as part of the treatment for patients with moderate to severe degrees of the disease.

Management approaches begin with, low-risk, and easily accessible patient-applied therapies, such as artificial tears for early-stage disease, and progress to more advanced therapies for more severe forms of DED [14].

Various pharmacological agents with a secretagogue effect can stimulate watery secretion, mucus secretion, or both. Topical diquafosol eye drops have been favorably evaluated in several clinical trials [5, 15, 16]. This agent is able to stimulate watery and mucous secretion in both animals and humans. It is also possible to administer orally cholinergic agonists, particularly pilocarpine and cevimeline, to treat severe DED. They have FDA-approved indications for the treatment of dry mouth associated with Sjögren’s syndrome [17, 18].

### How the intervention might work

Secretagogue therapy suggest advantages over using artificial tears. Diquafosol solution lowers the corneal fluorescein and rose Bengal scores compared to artificial tears [19] and improves the tear break time [20]. Another report suggested the superior efficacy and safety of 3% diquafosol ophthalmic solution compared to the use of other secretagogue therapies such as Cevimeline and pilocarpine [18, 21].

Regarding the long-term efficacy and safety of 3% diquafosol ophthalmic solution, the therapy was shown to significantly improve both subjective (dry eye symptom scores) and objective symptoms (eye staining score and tear function tests); the major adverse reactions were eye discharge, eye irritation, and eye pain; nevertheless the symptoms remitted after 28 days [22].

The use of acetylcholine analogs has been studied as a possible alternative for Sjögren’s syndrome; although most studies have shown more benefits for dry mouth, there is strong evidence that it can improve DED symptoms [23]. A possible disadvantage of muscarinic agonists is that they are contraindicated in angle-closure glaucoma and should be used with care in patients with asthma and heart disease [23].

### Why it is important to do this review?

DED is a condition that affects the ocular surface and affects millions of people around the world; the presentation of symptoms is very varied since it ranges from a foreign body sensation to severe pain [1, 4], which can make activities of daily living impossible. Buchholz et al. found that patients with severe DED, dialysis, and severe angina are willing to spend the same amount of time treating their illnesses [24].

It is estimated that the prevalence of DED ranges from 5 to 35% with a predominance of females and with a maximum peak at age 60 where the prevalence reaches 70% with a greater trend for the Asian population [1–5].

Therefore, it is important to evaluate the current evidence to compare the use of secretagogues with the use of artificial tears in DED, to determine the better option in terms of effectiveness and safety.

## Objectives

With this review, we intent to compare the effectiveness and safety of secretagogues versus artificial tears for dry eye. Our aim is to produce reliable and high-quality evidence on the efficacy of the use of secretagogues in the treatment of dry eye and thus contribute to the creation of recommendations that assist clinicians in the decision making-process.

## Research question

What is the effect on the Ocular Surface Disease Index of patients with dry eye treated with pilocarpine, cevimeline, or diquafosol compared to the use of artificial tears?

**Table:** 

**Population**	Adult (18 +) Sjögren’s syndrome Dry disease Keratoconjunctivitis sicca Aqueous tear deficiency
**Intervention**	Pilocarpine Cevimeline Diquafosol
**Comparison**	Artificial tears
**Outcomes**	Dry eye disease severity variation Quality of life Adverse event Clinical Improvement

## Methods

### Eligibility criteria

#### Participants

We will include trials where the study population comprises people 18 years old or older with dry eye disease. We will confirm that the clinical trials include participants 18 years old or older during the full-text review, we will verify the inclusion criteria and, when possible, we will review the registry of these clinical trials to ensure this inclusion criterion.

#### Interventions

The treatment group will be participants of clinical trials treated with pilocarpine, cevimeline, or diquafosol.

#### Comparator

The control group will be artificial tears.

#### Context

The studies will probably be carried out in an outpatient setting since the condition does not warrant in-hospital management.

#### Types of studies

We will include randomized controlled trials that compare secretagogues and artificial tears. Study inclusion will not be restricted on the basis of language or publication status.

### Types of outcome measures

We will search and extract information from all the time points reported in the clinical trials, then we will do a stratified analysis combining only information from clinical trials that share the same time points.

#### Critical outcome

- Change in patient-reported symptoms, evaluated by the Ocular Surface Disease Index (OSDI).

#### Important outcomes

- Change in dry eye disease signs, quantified by:
  -
    - ◦ Change in tear film stability (tear film break-up time)
    - ◦ Change in the staining of the ocular surface (rose bengal stain score)
    - ◦ Change in the staining of the ocular surface (fluorescein stain score)
    - ◦ Change in the production of aqueous tears (Schirmer test)
    - ◦ Change in Quality of life, evaluated by the VRQoL Score (Vision-Related Quality of Life)

#### Adverse outcomes

We will compare the proportion of adverse outcomes between treatment groups at any time. We will consider adverse outcomes as reported by included studies. Specific adverse outcomes of interest will include, but not be limited to the following:

- Eye discharge
- Eye irritation
- Eye pruritus
- Eye pain
- Conjunctivitis
- Foreign body sensation
- Blepharitis
- Allergic conjunctivitis.

### Databases and information sources

The search of information will include the following electronic databases for randomized controlled trials. There will be no language or publication year restrictions.

- Cochrane Central Register of Controlled Trials (CENTRAL) (which contains the CEV Trials Register) in the Cochrane Library (latest issue)
- PubMed (1948 to present)
- Scopus (2004 to present)
- LILACS (Latin American and Caribbean Health Science Information Database (1982 to present)
- US National Institutes of Health Ongoing Trials Register ClinicalTrials.gov
- World Health Organization (WHO)
- International Clinical Trials Registry Platform (ICTRP)

We will search the reference lists of eligible studies identified from the electronic searches for additional relevant trials that may not have been identified through the search strategy.

### Search strategy

The search strategy was designed with the highly sensitive Cochrane strategy to identify randomized clinical trials version that maximizes sensitivity [25] (37) and complemented with the Peer Review of Electronic Search Strategies (PRESS) guideline [26, 27].

### Data collection and analysis

#### Selection of studies

The study selection will be performed independently by pairs of review authors (GSR, AKPV); they will assess the titles and abstracts of articles identified through the literature search and will compare these against the inclusion criteria. Each article will be assessed independently by both authors and will be classified as either “definitely relevant,” “possibly relevant,” or “definitely not relevant.”

Covidence software will be used to manage the screening process [28]. Any disagreement will be resolved by a third review author (NKL).

Then, we will obtain the full-text copies of all studies classified as “definitely relevant” or “possibly relevant.” Each review author will independently assess each study for inclusion and will label it as either “include” or “exclude.” A third review author will resolve any disagreement. We will document the reason for exclusion of each study excluded after reviewing the full report in a “Characteristics of excluded studies” table. We will use Google Translate to assess studies written in languages other than English and Spanish. We will illustrate the study selection process in a PRISMA diagram.

### Data extraction and management

Pairs of review authors will independently extract data from the included studies using a data extraction form (Additional File 1) adapted from the Cochrane Eyes and Vision (CEV) data extraction form [29] developed by CEV and accessed via Covidence. A third review author will resolve any disagreements.

One review author will enter data into Review Manager 5 (RevMan 5) [30], and a second review author will verify the data entered.

We will extract data on the following items:

- Study details
  - ◦ Registry
  - ◦ Sponsorship source
  - ◦ Country
  - ◦ Trial setting
  - ◦ Registration
  - ◦ Sample size (number of included eyes)
  - ◦ Follow-up period
- Population
  - ◦ Inclusion criteria
  - ◦ Exclusion criteria
  - ◦ Group differences
  - ◦ Baseline characteristics
  - ◦ Participant characteristics: age, sex, etc.
  - ◦ Outcomes
- Effect measures: For continuous variables, mean, standard deviation, 95% confidence intervals, *p* value, and the number of participants for whom the outcome was measured; for dichotomous variables, the number of events and participants for whom outcome data were collected

When only graphical presentations are provided, two review authors (GSR, AKPV) will extract the data separately using Plot Digitizer 2.6.9 [31] software. Any disagreement will be resolved by a third review author (NKL).

### Assessment of risk of bias in included studies

The Cochrane Collaboration Risk of Bias 2 (RoB 2) tool will be used to assess the RoB in bias arising from the randomization process, due to deviations from intended interventions, due to missing outcome data, in measurement of the outcome, in selection of the reported result, and other potential sources of bias [32].

Two authors of the review will classify the risk of bias as either “low,” “high”, or “unclear” (insufficient information for assessment). A third review author will resolve any disagreement between review authors.

#### Measures of treatment effect

We will calculate mean difference (MDs) with 95% confidence intervals (CIs) for continuous measures (e.g., the Schirmer test value, TBUT) and risk ratios (RRs) with the corresponding 95% CIs for dichotomous outcomes to estimate effects (e.g., adverse events, effective rate). We will choose a cut-off for ordinal outcomes and measurement scales to handle them as binary data or treat them as continuous data, as appropriate (e.g., OSDI score > 12, TBUT < 10 seg, Schirmer < 5 mm).

#### Unit of analysis issues

The participant will be the primary unit of analysis whenever (a) only one eye per participant is enrolled in the trial or (b) two eyes of an individual are treated as a single unit after being administered the same treatment (e.g. pilocarpine, cevimeline, and diquafosol). For studies that enrolled both eyes of participant and in which the eye was the unit of analysis, we will document whether the trial had a within-person design and analyzed the data appropriately.

#### Dealing with missing data

We will contact corresponding authors by email to obtain missing data or data reported unclearly in the study reports. We will allow three weeks for study authors to respond and will use the available information whenever there is no response.

#### Assessment of heterogeneity

We will compare the participant characteristics, study interventions, and outcomes across trials to assess for clinical and methodological heterogeneity. We will use the *I*^2^ statistic, which estimates the proportion of variation in observed effects not due to chance, to identify inconsistency among trials; an *I*^2^ statistic value of greater than 50–90% will represent substantial heterogeneity [33]. Chi-square test statistics will be used to assess the statistical heterogeneity among estimates of effect size from the included studies.

#### Assessment of reporting biases

If 10 studies, or more, are included, we will visually inspect funnel plots of the intervention effect estimates for evidence of asymmetry to identify publication bias.

An asymmetric funnel plot may suggest small study effects, which could be the result of reporting bias, heterogeneity, or differences in the methodological quality of studies. We will assess selective outcome reporting as part of the “Risk of bias” assessment among individual studies.

### Data synthesis

If there is no substantial heterogeneity between studies (*I*^2^ ≤ 50%), we will use the fixed effects model to synthesis the data, and if we encounter heterogeneity greater than 50%, we will use a random effects model. If we deem a meta-analysis as inappropriate (e.g., less than 2 comparisons, unshared outcomes between studies, etc.), we will document the reasons and report findings from the individual studies narratively. The qualitative synthesis will provide data on the number of eyes, interventions, outcomes reported, time points, year, and country of the included studies.

### Subgroup analysis and investigation of heterogeneity

If sufficient data is available from included studies, we will examine findings by the degree of DED severity at baseline among the study participants.

#### Sensitivity analysis

Where possible, we will perform sensitivity analyses for critical and important outcomes to explore the effects of restricting our analyses to trials judged to have adequate allocation concealment, adequate masking of outcome assessors, and had at least 80% follow-up of participants in each group, to determine the robustness of the conclusion.

### Strength of evidence

We will summarize the findings of the review using the Grades of Recommendation, Assessment, Development, and Evaluation (GRADE) approach; ratings for randomized controlled trials will be presented to form judgments regarding the certainty of the evidence within the text of the review. We will use the GRADEpro GDT software procedures and guidelines [34]. Assessment of GRADE evaluation will be conducted by two authors of the review (GSR, AKPV); footnotes will be used to justify decisions to downgrade or increase the ratings. Any disagreement will be resolved by a third review author (NKL).

### Protocol amendments

Protocol modifications will be reported both in PROSPERO and in the review publication.

## Discussion

DED presents with a broad spectrum of presentation that ranges from eye discomfort to ocular pain with blurry vision, affecting quality of life and limiting activities of daily living.

Randomized controlled clinical trials have been published suggesting the use of secretagogues such as pilocarpine, cevimeline, and diquafosol for the treatment of this disease. This systematic review aims to determine the efficacy and safety of the secretagogues pilocarpine, cevimeline, and diquafosol to help clinicians in the decision-making process.

### Supplementary Information

**Additional file 1. **Data extraction form.

**Additional file 2. **Personal cover letter

## Data Availability

Not applicable.

## Appendix

### Search strategy

PubMed

#1 ((randomized controlled trial[pt]) OR (controlled clinical trial[pt]) OR (randomized[tiab] OR randomized[tiab]) OR (randomly[tiab]) OR (trial[tiab]) OR (groups[tiab])) NOT (animals[mh] NOT humans[mh]).

#2 (Dry eye [tw] OR Dry eye syndrome [tw] OR Dry eye disease [tw] OR Conjunctivitis sicca [tw] OR Keratoconjunctivitis sicca [tw] OR Keratitis sicca [tw]).

#3 (Pilocarpine [pa] OR Cevimeline [pa] OR Diquafosol [pa]) OR (Pilocarpine [tiab] OR Cevimeline [tiab] OR Diquafosol [tiab]).

#4 (Artificial tear [tiab] OR Ocular lubricant [tiab]) OR (Artificial tear [pa] OR Ocular lubricant [pa]).

#5 #1 AND #2 OR #3 OR #OR

Cochrane Central Register of Controlled Trials (CENTRAL).

#1 MeSH descriptor: [Dry Eye] explode all trees.

#2 MeSH descriptor: [Dry Eye Disease] explode all trees.

#3 MeSH descriptor: [Dry Eye Syndrome] explode all trees.

#4 (Pilocarpine):kw.

#5 (Cevimeline):kw.

#6 (Diquafosol):kw.

#7 (Artificial tear):kw.

#8 (Ocular lubricant):kw.

#9 #1 OR #2 OR #3 OR #4 OR #5 #6 OR #7 OR #8

LILACS

(TW:”randomized controlled trial” OR TW:”controlled clinical trial” OR TW:randomized OR TW:randomized OR TW:randomly OR TW:trial OR TW:groups) AND ((TW:”Dry eye” OR TW:”Dry eye syndrome” OR TW:”Dry eye disease” OR TW:”Conjunctivitis sicca” OR TW:”Keratoconjunctivitis sicca” OR TW:”Keratitis sicca”) OR (TW:Pilocarpine OR TW:Cevimeline OR TW:Diquafosol) OR (TW:”Artificial tear” OR TW:”Ocular lubricant”)).

ClinicalTrials.gov

((Dry eye OR Dry eye syndrome OR Dry eye disease OR Conjunctivitis sicca OR Keratoconjunctivitis sicca OR Keratitis sicca) OR (Pilocarpine OR Cevimeline OR Diquafosol) OR (Artificial tear OR Ocular lubricant)).

WHO ICTRP

((Dry eye OR Dry eye syndrome OR Dry eye disease OR Conjunctivitis sicca OR Keratoconjunctivitis sicca OR Keratitis sicca) OR (Pilocarpine OR Cevimeline OR Diquafosol) OR (Artificial tear OR Ocular lubricant)).

Scopus

((randomized AND controlled AND trial) OR (controlled AND clinical AND trial) OR (randomized OR randomized) OR (randomly) OR (trial) OR (groups)) AND (dry AND eye OR dry AND eye AND syndrome OR dry AND eye AND disease OR conjunctivitis AND sicca OR keratoconjunctivitis AND sicca OR keratitis AND sicca) AND (pilocarpine OR cevimeline OR diquafosol) OR (artificial AND tear OR ocular AND lubricant).

## Abbreviations

DED: Dry eye disease
OSDI: Ocular Surface Disease Index
RoB: Risk of bias
TBUT: Tear break-up time
VRQoL: Vision-Related Quality of Life
RevMan 5: Review Manager 5
CEV: Cochrane Eyes and Vision
MDs: Mean difference
CIs: Confidence intervals
RRs: Risk ratios
GRADE: Grading of Recommendation, Assessment, Development and Evaluation
PRESS: Peer Review of Electronic Search Strategies
