# Supplementary material for: Safety and efficacy of pilocarpine, cevimeline, and diquafosol compared to artificial tears for the treatment of dry eye: protocol for a systematic review
Source: Syst Rev. 2022 May 28;11:105. doi: 10.1186/s13643-022-01979-4 (PMC9148479; doi:10.1186/s13643-022-01979-4)
Supplement: Supplementary file 1 — Additional file 1. Data extraction form. [file 13643_2022_1979_MOESM1_ESM.docx]

**Data extraction form.**

**Methods**

| **Mandatory** | **Optionals** |
| --- | --- |
| Study Design. | 1. Exlusion after randomization. 2. Loss of follow-ups. 3. Randomized number. 4. Number analized. 5. How was missing information handled? 6. Reported power calculation (yes/no). If yes, mention sample size and power. 7. If there were any setbacks. |
| 1. Randomization of people for treatment. 2. Individual eye randomization. 3. Randomization of communities to treatment. 4. Other: specify. |  |
| Unit of Analysis |  |
| 1. Inclusion of one eye. Describe how the inclusion was made. 2. Both eyes included in the same treatment. Specify how they were selected for inclusion.   Define how data analysis was performed (best/worst/average/ both adjusted for within-person correlation/ both not adjusted for within person correlation.)   1. Both eyes included but it different treatments. Specify whether a pair matching analysis was performed. |  |

**Participants.**

| **Mandatory** | | **Optionals** |
| --- | --- | --- |
| 1. Country. 2. Total number of participants. 3. Number (%) of men. 4. Number (%) of women. 5. Average age. 6. Age range. 7. Inclusion criteria. 8. Exlusion criteria. | This information must be reported in all included patients in the study and not only from analyzed population.  If only analyzed population is included, it should be mentioned that way. | 1. Ethnic group. 2. Basal characteristics. 3. Enviroment. |

**Interventions**

| **Mandatory** | |
| --- | --- |
| Study Participants Assigned to Intervention. | 1. Number of randomized people in this group. 2. Drug/ intervention name. 3. Dose. 4. Frequency. 5. Route of administration. |
| Study Participants Assigned to Comparison. | 1. Number of randomized people in this group. 2. Drug/ intervention name. 3. Dose. 4. Frequency. 5. Route of administration. |

**Outcomes**

| **Mandatory** | **Optionals** |
| --- | --- |
| - Primary and secondary outcomes as defined in the study protocol. | - Planed follow up versus real follow up. |
| 1. Outcomes list. 2. Report of adverse effects (yes/no). 3. Treatment duration/ follow up. 4. Intervals at which the results are going to be evaluated. |  |
