# Supplementary material for: Safety and efficacy of pilocarpine, cevimeline, and diquafosol compared to artificial tears for the treatment of dry eye: protocol for a systematic review
Source: Syst Rev. 2022 May 28;11:105. doi: 10.1186/s13643-022-01979-4 (PMC9148479; doi:10.1186/s13643-022-01979-4)
Supplement: Supplementary file 2 — Additional file 2. Personal cover letter [file 13643_2022_1979_MOESM2_ESM.docx]

**PERSONAL COVER LETTER**

Dry eye is a condition that affects the ocular surface and affects millions of people around the world. It is estimated that the prevalence of dry eye ranges from 5% to 35% with a predominance in females and with a maximum peak. at age 60 where the prevalence reaches 70% with a higher trend for the Asian population (1–5). The symptoms are very varied, ranging from a foreign body sensation to severe pain, which can make daily activities impossible (1,4).

A scheme for the treatment of dry eye has been proposed, with the use of artificial tear being the mainstay of treatment (6). In this scheme, the use of secretagogues is suggested as part of the treatment for patients with moderate to severe degrees. Although the use of secretagogues has been proposed for the treatment of dry eye and clinical trials have recently been published in which the evidence suggests a benefit in the use of this pharmacological group (7–12), a systematic review and meta-analysis have not been published that allows us to evaluate whether the efficacy is constant in different populations, as well as the magnitude of the effect associated with these drugs.

Our research question is what is the effect on the ocular surface disease index of patients with dry eye treated with Pilocarpine, Cevimeline and Diquafosol compared to the use of artificial tears without preservatives? The primary objective is to evaluate the efficacy of dry eye patients treated with secretagogues.

This systematic review is pertinent, since producing reliable and high-quality evidence on the efficacy of the use of secretagogues in the treatment of dry eye will contribute to the creation of recommendations and clinical practice guidelines.

The protocol has been registered on the PROSPERO platform, and we consider BMC systematic reviews as the adequate journal for this protocol. The content of the manuscript has not been published, or submitted for publication elsewhere, all authors have approved the manuscript for submission and none of the research participants has declared to have any conflict of interest

**REFERENCES**

1.	Schaumberg DA, Nichols JJ, Papas EB, Tong L, Uchino M, Nichols KK. The international workshop on meibomian gland dysfunction: Report of the subcommittee on the epidemiology of, and associated risk factors for, MGD. Investig Ophthalmol Vis Sci. 2011;52(4):1994–2005.

2.	Nichols KK. The international workshop on meibomian gland dysfunction: Introduction. Investig Ophthalmol Vis Sci. 2011;52(4):1917–21.

3.	Daniel Nelson J, Shimazaki J, Benitez-del-Castillo JM, Craig J, McCulley JP, Den S, et al. The international workshop on meibomian gland dysfunction: Report of the definition and classification subcommittee. Investig Ophthalmol Vis Sci. 2011;52(4):1930–7.

4.	Clayton JA. Dry eye. N Engl J Med. 2018;378(23):2212–23.

5.	Dana R, Bradley JL, Guerin A, Pivneva I, Stillman IÖ, Evans AM, et al. Estimated Prevalence and Incidence of Dry Eye Disease Based on Coding Analysis of a Large, All-age United States Health Care System. Am J Ophthalmol. 2019;202:47–54.

6.	Rauz S, Koay S-Y, Foot B, Kaye SB, Figueiredo F, Burdon MA, et al. The Royal College of Ophthalmologists guidelines on serum eye drops for the treatment of severe ocular surface disease: full report. Eye. 2017;1–16.

7.	Fukuoka S, Arita R. Tear film lipid layer increase after diquafosol instillation in dry eye patients with meibomian gland dysfunction: a randomized clinical study. Sci Rep. 2019;9(1):1–11.

8.	Shimazaki J, Seika D, Saga M, Fukagawa K, Sakata M, Iwasaki M, et al. A Prospective, Randomized Trial of Two Mucin Secretogogues for the Treatment of Dry Eye Syndrome in Office Workers. Sci Rep. 2017;7(1):1–10.

9.	Koh S, Ikeda C, Takai Y, Watanabe H, Maeda N, Nishida K. Long-term results of treatment with diquafosol ophthalmic solution for aqueous-deficient dry eye. Jpn J Ophthalmol. 2013;57(5):440–6.

10.	Ono M, Takamura E, Shinozaki K, Tsumura T, Hamano T, Yagi Y, et al. Therapeutic effect of cevimeline on dry eye in patients with Sjögren’s syndrome: A randomized, double-blind clinical study. Am J Ophthalmol. 2004;138(1):6–17.

11.	Jun I, Choi S, Lee GY, Choi YJ, Lee HK, Kim EK, et al. Effects of Preservative-free 3% Diquafosol in Patients with Pre-existing Dry Eye Disease after Cataract Surgery: A Randomized Clinical Trial. Sci Rep [Internet]. 2019;9(1):1–10. Available from: http://dx.doi.org/10.1038/s41598-019-49159-0

12.	Park CH, Lee HK, Kim MK, Kim EC, Kim JY, Kim TI, et al. Comparison of 0.05% cyclosporine and 3% diquafosol solution for dry eye patients: A randomized, blinded, multicenter clinical trial. BMC Ophthalmol. 2019;19(1):1–10.
